# Supplementary material for: Exploring factors contributing to patient decision-making in the care journey to elective hernia care in Kenya
Source: PLoS One. 2025 Nov 20;20(11):e0337430. doi: 10.1371/journal.pone.0337430 (PMC12633918; doi:10.1371/journal.pone.0337430)
Supplement: S2 File — (DOCX) [file pone.0337430.s002.docx]

In this Supporting File 2 (S2 File), we present semi-structured interview guide which was used for all interviews. At the initiation, the interviewer SN introduced herself, explained the objectives of the interview, and confirmed patient consent to interview recording. Following consent, SN interviewed patients who were prior to discharge using the “discharge interview” section, and patients who were attending their post-discharge follow-up clinic visiting using the “follow-up interview” section. For patients who were only able to attend one interview, SN combined the two interview scripts into one session. All probes following questions were to encourage further patient discussion and used as appropriate.

**Discharge Interview**

***Opening questions***

1. Can you tell us about yourself and what you do for work?
   1. Probe 1: Can you describe your activities in a day?
2. Walk me through the time when you first noticed your condition?

***Hernia impact***

1. Can you describe how this problem has affected your day-to-day life?
   1. Probe 1: How has this problem affected how you can perform activities day-to-day?
2. Can you tell me more about when you decided to see someone for this problem?
   1. Probe 1: Was there some new change or some event that made you think you should see a doctor?
3. Who did you go see first for this problem?
   1. Probe 1: Why did you decide to go see this person/go to this place?

***Hernia diagnosis***

1. How did you feel when receiving this diagnosis of hernia?
   1. Probe 1: Did you have any worries or fears? If yes, can you tell me why?
2. Can you describe for me what a “hernia” is in your own words?
   1. Probe 1: How did you learn what you know about hernias?
3. Can you share with me any experience you have had with hernias outside of your own diagnosis?
   1. Probe 1: Have you known many people with hernias?

***Seeking care***

1. Can you walk me through how you have arrived at MTRH for your hernia management?
   1. Probe 1: Why did you choose to come to MTRH instead of another facility?
   2. Probe 2: If you were referred here, who referred you? Were they a surgeon?
2. Can you tell me more about how you felt when you were told you needed to go to theatre for an operation?
   1. Probe 1: Any worries or concerns?
3. Can you describe any actions you took at home to prepare for your surgery?
   1. Probe 1: How about changes at home?
   2. Probe 2: How about preparing for finances?

***Receiving care***

1. How did you feel after your operation?
2. Can you tell me what you understand about what was done in your operation?
   1. Probe 1: Is there information you wish to know?
3. Can you tell me, what you understand about what the doctors say when they visit you? Probe 1: Did you get your questions answered by the doctor each day?

***Discharging home***

1. Can you tell me how you feel now that you have been cleared for discharge?
   1. Probe 1: Do you have any questions or concerns about going home?
2. Can you tell me in your own words what instructions you need to follow during your recovery period?

**Follow-up Interview**

1. Can you introduce yourself and tell us when you had your hernia surgery?

***Surgery recovery***

1. Can you describe how your recovery is at home?
   1. Probe: What has been going well?
   2. Probe: What has been going poorly?
2. Do you have any remaining symptoms that bother you? Which ones?
   1. Probe: How much discomfort have these symptoms caused you?
3. In your own words, what instructions do you remember receiving before you were cleared for home?
   1. Probe: How long were you told to avoid lifting for?
   2. Probe: What is the most weight you were told you could lift?
4. How were you able to follow these instructions at home?
5. Did anything happen that you did not expect during your recovery?

***Return of function***

1. Can you describe your day-to-day activities now during recovery?
   1. Probe: How soon did you resume your activities after the procedure?
2. In what ways has your family supported you in your recovery?
   1. Probe: Can you describe what activities they may help you with?
3. How long remaining before you think you will be fully healed?

Now I would like to refer back to the map you provided before **[use map]**

***Impact of surgery***

1. Looking back on this map can you describe what challenges you faced?
   1. Probe: What about before the operation?
   2. Probe: What about after the operation?
   3. Probe: What about at home?
2. How did getting this repair changed your life?
   1. Probe: How is your life different now than it was when you had a hernia?
3. Can you tell me about any time when you faced financial challenges during your journey?
   1. Probe 1: How did you address them?
4. Can you tell me anything you would change about your journey if you were able?

***Impression of surgery***

1. If you find someone with the same problem, would you recommend this surgery to other people?
   1. Probe: What would you tell other people about hernias now after your operation?
2. Was anything surprising about the care you received compared to your expectations?
