# Supplementary material for: Exploring factors contributing to patient decision-making in the care journey to elective hernia care in Kenya
Source: PLoS One. 2025 Nov 20;20(11):e0337430. doi: 10.1371/journal.pone.0337430 (PMC12633918; doi:10.1371/journal.pone.0337430)
Supplement: S3 Table — (DOCX) [file pone.0337430.s003.docx]

**S3 Table 1: Patients desire power over care: Theme definition, subthemes, and representative quotes.**

| **Power is transferred as consent or knowledge**:  Patients desire power to manage their care. Loss of power to control symptoms initiates the need to transfer power to providers through consent. Providers can re-empower patients through providing correct knowledge regarding their condition. | |
| --- | --- |
| ***Subtheme*** | ***Representative Quote*** |
| Patients desire power over health | “I used to take medication, I used to go to the chemist and ask for the same medicine each time, it normally reduced the pain and reduced the swelling and everything would be alright” (*ID28, male, 26 years, inguinal*) |
| Escalating symptoms inspire transfer of power via consent | “I was in so much pain, and I couldn’t do anything about it. The size it had swollen to was the largest I had ever seen. I decided to share it with two of my friends, they took me to a chemist and that’s when they told me to go to Referral” (*ID28, male, 26 years, inguinal*)  “It reached a point where I couldn’t do any housework. I used to feel like my chest was full and I used to strain while breathing. I also had a feeling of fullness in my stomach… it was really painful” (*ID16, female, 55 years, epigastric*)  “From September to October [it] became unbearable because I could not stand up for long, the pain increased…that’s why I came to MTRH” (*ID12, male, 53 years, inguinal*)  “When the pains starts, you can’t eat, you go to the toilet just vomiting, and it makes you diarrhea…the pain was very persistent and severe. That’s when I went to see a doctor” (*ID14, female, 70 years, epigastric*) |
| Patients regret lacking adequate knowledge | “Before I knew what the problem was, I was not bothered because I thought it’s just a normal sickness. As the pain was coming, I take medicine then it disappears. But when I was told about it, I was very disturbed” (*ID17, female, 56 years, epigastric*)  “I can say, missing information, not being told, or…not doing any scanning or research… I used to live in pain not knowing what it was… if I knew what it was earlier I would have had it taken care of. I wouldn’t have suffered for that long” (*ID28, male, 26 years, inguinal*) |
