# Supplementary material for: Exploring factors contributing to patient decision-making in the care journey to elective hernia care in Kenya
Source: PLoS One. 2025 Nov 20;20(11):e0337430. doi: 10.1371/journal.pone.0337430 (PMC12633918; doi:10.1371/journal.pone.0337430)
Supplement: S4 Table — (DOCX) [file pone.0337430.s004.docx]

**S3 Table 2: Trust is the currency for engagement: Theme definition, subthemes, and representative quotes.**

| **Trust is the currency for engagement:**  Trust is a limited resource and is required for patient engagement and retention in care. Satisfaction with prior experience or influence from trusted contacts can result in gain or loss of trust | |
| --- | --- |
| ***Subtheme*** | ***Representative Quote*** |
| Trusted family/community influence patient trust | “The reason which mostly made me choose here was because the chemists that I used to go to knew that the best place for treatment was here in Referral. They had trust in this place. They were the ones who advised me to come here. I didn’t know if there was any other place where I could be treated, so I followed their instructions which said I need to come here” (*ID28, male, 26 years, inguinal*)  “I heard [MTRH] praises and most of my relatives who live here told me that is was the best hospital to visit in my case” (*ID17, female, 56 years, epigastric*) |
| Trust aligns with satisfaction in care | “The difference between this referral hospital and the other one is that I never saw the surgeon [there]…unlike here, I see the surgeon whenever he checks on you, and I see that the differences are so good” (*ID30, male, 66 years, incisional*)  “[At this hospital], you will get some advice, [and] they help you. Even if you are sick, you feel like you are good since there are some people who come to talk to you…hence you get healed” (*ID16, female, 55 years, epigastric*)  “The person who admitted me was kindhearted. I was admitted on 28th and I was to go for surgery on 29^th^, they really attended me very well…I felt I was in safe hands and I’m grateful” (*ID17, female, 56 years, epigastric*) |
| Adequate trust promotes consent to care and lowered anxiety | “I did not have any anxiety. I got rid of the anxiety when I was told that I would get help and recover” (*ID19, male, 81 years, epigastric*)  “No there was nothing I would want to ask. What would I ask? I did not study that profession. There are professionals in every area” (*ID19, male, 81 years, epigastric*)  “I didn’t fear [about surgery]. I told them to come to take me fast, and I recovered” (*ID20, female, 73 years, epigastric*) |
| Inadequate trust leads to patient disengagement | “We went to Kapsabet but they were really slow. Sometimes they would misplace your file and start looking for it. That’s when my son told me to go to Referral” (*ID16, female, 55 years, epigastric*)  “I came here from Pivot. They were not treating me well. I had some pains they were not following up on, so they would tell you to wait, the doctor is the only one. So that’s why I came [to MTRH] because I saw they are treating me well” (*ID22, male, 46 years, inguinal*) |
