# Supplementary material for: Exploring factors contributing to patient decision-making in the care journey to elective hernia care in Kenya
Source: PLoS One. 2025 Nov 20;20(11):e0337430. doi: 10.1371/journal.pone.0337430 (PMC12633918; doi:10.1371/journal.pone.0337430)
Supplement: S5 Table — (DOCX) [file pone.0337430.s005.docx]

**S3 Table 3: Context of healthcare environment remains foundation for accessing care: Theme definition, subthemes, and representative quotes.**

| **Context is the foundation for care**  Context, defined by both internal patient factors and external healthcare environment factors, provides the foundation required to make patient-provider care activities possible. | |
| --- | --- |
| ***Subtheme*** | ***Representative Quote*** |
| Patients willing to sacrifice for ideal care | “People come from different regions to come to referral where treatment is…knowing they’ll get treated here” (*ID1, female, 61 years, incisional*)  “At that time, I had 1500 which we used to pay [for insurance enrollment]. That’s when I gave myself courage and said to myself that paying would be of help than using my own money, yet the expense is high” (*ID19, male, 81 years, epigastric*)  “Sometimes when they are telling you to take you to the theater you don’t know I’ve been there before you don’t have any options, you just have to go…we had to look for funds and you sell sheep so that you can look for money” (*ID30, male, 66 years, incisional*)  “We came to the hospital 3 times and when they told me, that she will undergo operation, she was admitted in ward 5” (*ID20, female, 73 years, epigastric*)  “There difficulty is there because of the transport, returning from the trip, tiredness then you are sent back home without treatment” (*ID23, male, 42 years, epigastric*)  “I had a hard time getting here. I was trying to get support from family members” (*ID4, male, 57 years, inguinal*) |
| Ideal care is close, affordable, quality and efficient | “At home there is another hospital near the lake some people go there they get X-Ray and they are sent here and when they get here they will still get another X-Ray. So, I decided to come here where I can get all of them at once” (*ID19, male, 81 years, epigastric*)  “I went to [county hospital] where they told me to come back on [a later] date. So I went to a private [hospital]…near my home” (*ID16, female, 55 years, epigastric*)  “I went to the chemist to get some medicine and stayed for almost a year…it is closer to where I live. It’s within walking distance” (*ID28, male, 26 years, inguinal*) |
| Patients prioritize health | “I called to ask for rescheduling of my appointment to the following week since I was tending to my husband in the ward…unfortunately, he passed on shortly after. As I was coming for my appointment he was in the mortuary, and I asked God for strength as my health is also important” (*ID1, female, 61 years, incisional*) |
| Patients access to ideal care limited by internal personal limitations | “I came to Referral because of the pain …I didn't go to other hospitals because of the money I didn't have” (*ID8, male, 31 years, inguinal*)  “There was another doctor when I went for clinic. He checked my stomach and felt as if I had swelled. I had not had in NHIF that time… I would have gone to the other [private] hospital” (*ID22, male, 46 years, inguinal*) |
| Financially vulnerable patients dependent on public hospitals as accessible care option | “I went to a private hospital but we had a conflict of interest due to the cost. I thought I would get assisted quickly [at the private hospital, but]… they were asking for an amount that I couldn’t get so I decided to move to Eldoret” (*ID3, male, 74 years, incisional*) |
| Patients access to ideal care limited by external systemic limitations | “No, there wasn’t any hospital that weren’t able to help me, I just came here. The only thing about the other hospital was that they didn’t have the scanning machine” (*ID5, male, 78 years, inguinal*)  “I have had [the hernia] for long. I also went to a lot of hospitals where I was told they don’t know what it was” (*ID17, female, 56 years, epigastric*)  “No, they did not diagnose me, they gave me some painkillers. And they told me that they don’t have equipment for [diagnosing]” (*ID9, male, 63 years, inguinal*) |
