# Supplementary material for: Exploring factors contributing to patient decision-making in the care journey to elective hernia care in Kenya
Source: PLoS One. 2025 Nov 20;20(11):e0337430. doi: 10.1371/journal.pone.0337430 (PMC12633918; doi:10.1371/journal.pone.0337430)
Supplement: S5 File — (DOCX) [file pone.0337430.s007.docx]

Supplemental File 5 (S5_File): Codebook utilized in qualitative analysis

| CODE | CARE PHASE | SUB-CODE | DEFINITION |
| --- | --- | --- | --- |
| Patient understanding of hernia | Seeking |  | Use when patients communicate knowledge about hernia disease PRIOR to current interaction with/education by medical providers. For recurrent hernias, treat recurrence as new diagnosis and refer to knowledge prior to engaging with providers on current admission for recurrent hernia. Do NOT use when referring to newly gained information following interaction with current health providers. |
| Patient desire more information… | Seeking |  |  |
|  |  | About hernia | Use when patients express desire for specific information about HERNIA. Do NOT use when patient express general fear or doubt |
|  |  | About surgery | Use when patients express desire for specific information about SURGERY. Do NOT use when patient express general fear or doubt |
| Patient delays to care | Seeking |  |  |
|  |  | Fear of disease | Use when fear of DISEASE (unknown OR incorrect assumptions) DELAYS patients in seeking care. Do NOT use when patient express fear of TREATMENT/CARE (see: “neg emotions: Fear”) |
|  |  | Shame with hernia delays care | Use with specific examples of when patient express shame from having visible hernia swelling which DELAYS care due to desire for privacy and lack of communication with others |
|  |  | Lack knowledge | Use when patients do not have adequate knowledge to guide care seeking decisions, and therefore put it off, causing delays |
|  |  | Self-treatment | Use when patient attempting to manage hernia symptoms themselves rather than seeking formal care, resulting in DELAY of care seeking onset |
| Living with hernia | Seeking |  |  |
|  |  | Patients managing multiple medical issues simultaneously | Use when patients report other medical needs which they have while they are pursuing care for hernia. May require them to attend multiple different care clinics |
|  |  | Hernia limiting function | Use when patients report loss of ability to perform daily tasks (ex: work, chores, social activities) which they would otherwise participate in if hernia was not present. Do NOT use when patient referencing inability to be present for tasks due to need to attend clinic visits (see: “patient willing to sacrifice for health”) |
|  |  | Loss formal employment | When patient lose formal employment due to functional limitations from hernia being present. DO NOT USE when referring to limitations in everyday activities at home |
|  |  | Patients live with symptoms for long time | Use when patients report experiencing hernia symptoms over a long period |
|  |  | Fluctuating symptoms of hernia | Use when patient describe on-and-off nature of hernia symptoms. DO NOT use when referring to severe symptoms which led to seek care (see: “inability to control symptoms leads to care seeking”) |
|  |  | Recurrent hernia | Use when patients report prior history of hernia repair, and are now experiencing recurrent hernia requiring repeat care |
|  |  | Balancing family responsibilities with self-care | Use when patients report how they must manage family responsibilities during care seeking |
| Risk factors for hernia | Seeking |  |  |
|  |  | Cough | Use when patients report having cough as a potential risk or source for hernia development. |
|  |  | Exertion | Use when patients report having exertion as a potential risk or source for hernia development |
|  |  | Pregnancy | Use when patients report pregnancy as a potential risk or source for hernia development |
| Passive participation in care | Seeking, reaching |  |  |
|  |  | View doctors as professionals who should be trusted | Use in instances when patients receive care as given WITHOUT questioning providers, even though they may have questions. Trust stems from view of providers as professionals who received training/have expertise to provide care which patients don't have, and thus should not be questioned |
|  |  | Trust in providers reduce fear/anxiety | Trust stems from desire to be taken care of by providers, which also helps to reduce patient anxieties. Patients able to select provider to trust as one way to gain control over care |
|  |  | Gain information that hernia is simple problem | Use when patients learn that their hernia is a simple problem that is easily solved. May received info from trusted family/community OR providers debunking incorrect information |
| Active participation in care | Seeking, reaching |  |  |
|  |  | Patient advocate for self | Use in instances when PATIENTS question care or advocate for their needs; may have conflict with providers or care which is offered |
|  |  | Self-referral | Use in examples when patient describes seeking care at center OUTSIDE of that which was recommended by providers |
|  |  | Family/community advocate for patients | Use in instances when non-medical FAMILY/COMMUNITY question care or advocate for their needs; may have conflict with providers. |
| Tipping point | Seeking, reaching |  |  |
|  |  | Visible swelling prompts care seeking | Use when patients report a shift in care seeking behavior due to seeing visible hernia swelling which was not present before. May be driven by shame, symptoms, or fear |
|  |  | Inability to control symptoms leads to care seeking | Use when patients who lived with symptoms for a long time and are no longer able to control symptoms as they have been able to prior, thus prompting care seeking. Do NOT use when involving provider care; should only refer to patient self-management |
|  |  | Ineffective treatment inspires departure from formal provider recommendations/referrals | Use when patients had been following provider recommendations regarding referrals until a negative experience involving inadequate care (ex: misdiagnosis/inadequate control symptoms) causes patients to change care seeking activity (ex: self-referral, delay care, taking medications, etc). Do NOT use when involving patient self-management only |
|  |  | Fear of disease | Use when fear of DISEASE (unknown OR incorrect assumptions) DRIVES patients to seek care. Do NOT use when patient express fear of TREATMENT/CARE (see: “neg emotions: Fear”) |
| Patients have many options for healthcare services | Reaching |  | Use when patients explore different health centers to address different problems (ex: attend regional centers for smaller (non-surgical) issues) |
| Others influencing care seeking | Reaching |  |  |
|  |  | Family/community influence (non-medical) | Use when patients report how outside factors, specifically non-medical FAMILY/COMMUNITY, guided their care seeking decisions |
|  |  | Healthcare provider influence | Use when patients report how outside factors, specifically MEDICAL PROVIDERS, guided their care seeking decisions |
| Perceive MTRH as best care | Reaching |  |  |
|  |  | MTRH intrinsically better | Use when patients report belief that MTRH is the highest quality standard of care BEFORE they receive any care there. Do NOT use when expressing satisfaction with the care already received at MTRH (see: “satisfied with care/attention at MTRH”) |
|  |  | Collected care resources at MTRH provides desired efficiency | Examples of patients preferring to come to MTRH where all needs/resources/infrastructure may be met here compared to needing to attend many separate referrals/visits at lower levels of care |
|  |  | More understanding of problems at MTRH | Use when patients express willingness to accept challenges experienced at MTRH because they perceive that MTRH care is the best, even in the face of experienced challenges. Do NOT use when only expressing MTRH is best care with NO experience of challenges suggesting otherwise (see: “MTRH intrinsically better”) |
| Perceive regional centers as inadequate | Reaching |  |  |
|  |  | Inadequate care | Use when patients report receiving inadequate care (ex: inadequate treatments, unavailable resources, etc) when seeking care at REGIONAL centers |
|  |  | Inadequate attention | Use when patients report receiving inadequate attention (ex: long wait times, little interaction/care from providers, etc) when seeking care at REGIONAL centers |
| Desired features in care | Reaching |  |  |
|  |  | Efficiency | Use when patients express desire for care which is provided promptly |
|  |  | Close distance | Use when patients express desire for care which is close to home/in convenient location |
|  |  | Attention from providers | Use when patients express desire for care which involves close attention from providers. May be in the form of time, communication, education, etc |
|  |  | Affordable | Use when patients express desire for care which is affordable without causing excessive negative impacts on patient financial status |
| Patient gains hernia knowledge | Reaching |  |  |
|  |  | Gain understanding of hernia risk factors | Use when patients can communicate new CORRECT knowledge gained from interactions with PROVIDERS regarding hernia DISEASE (risk factors, symptoms). Do NOT use when referring to information gained from family/non-providers |
|  |  | Gain understanding of need for surgical repair of hernia | Use when patients can communicate new CORRECT knowledge gained from interactions with PROVIDERS regarding hernia TREATMENT (ex: requires surgical repair). Do NOT use when referring to information gained from family/non-providers |
|  |  | Incorrect information gained | Use when patients can communicate new INCORRECT knowledge gained from interactions with PROVIDERS regarding hernia disease or management. Do NOT use when referring to information gained from family/non-providers |
| Patient willing to sacrifice for health | Reaching |  |  |
|  |  | Patients persist in care seeking in face of challenges | Use when patients express the challenges and barriers they had to overcome when navigating through care journey. Do NOT use when referring to disability/decreased function due to presence of hernia (see: “hernia limiting function”) |
|  |  | Take time for recovery | Use when patient express needing adequate time to achieve recovery and are willing to take that time fully. Sometimes even beyond what is recommended by providers |
|  |  | Money | Use when patients report using funds towards hernia care instead of for other causes; often requiring sacrifice or inconvenience to patient/family |
|  |  | Willing to travel far for health | Use when patients report travelling far to get to hernia care; often requiring sacrifice or inconvenience to patient/family |
| Financial delays to care | Reaching, receiving |  |  |
|  |  | Lack funds to initiate care | Use when patients report inadequate financial ability to pay for needed care or to initiate care |
|  |  | NHIF fees are high | Use when patients report inadequate financial ability to enroll (or reactivate) in NHIF which would cover medical costs. |
|  |  | NHIF activation after problem present | Use when patient express delay from needed to wait for NHIF activation since enrollment occurred after need for surgery is identified |
|  |  | Non-medical costs are significant | Examples of NON-medical costs which patients still had to pay outside of insurance coverage (ex: travel to clinic). DO NOT use when referring to medical bills alone |
|  |  | Financial challenges when no NHIF | Use when patient describing challenges paying for care when don't have NHIF. Do not use if patient is describing NHIF fees being unaffordable (see: “NHIF fees high code”) |
| Negative emotions | Reaching, receiving |  |  |
|  |  | Fear/feelings of isolation/loneliness | Use when expressing negative experiences of patients regarding feelings of loneliness or isolation while receiving care for hernia. Do NOT use when referring to living with stigma PRIOR to receiving care (see: “delays: privacy”). Do NOT use when referring to providers not having time to see them (see: “dissatisfied with care at MTRH”) |
|  |  | Fear/feelings of loss of control | Use when expressing negative experiences of patients regarding feelings of loss of control or vulnerability associated with receiving hernia care. Do NOT use when referring to loss of control with hernia outside of care receiving (see: “living with hernia”) |
|  |  | PRE-operative fear of poor care outcomes | Use when expressing negative experiences of patients regarding feelings of fear or doubt regarding poor outcomes PRIOR to surgical repair. Do NOT use with regards to fear involving hernia diagnosis or symptoms (see: “delays: fear”) |
|  |  | Fear of POST-operative poor care outcomes | Use when expressing negative experiences of patients regarding feelings of fear or doubt regarding poor outcomes FOLLOWING surgical repair. |
|  |  | NHIF not adequate coverage | Use when expressing negative experiences of patients regarding NHIF not being sufficient to cover medical bills. DO NOT use when referring to additional costs outside medical bills (see: “non-medical costs are significant”) |
| Positive emotions | Reaching, receiving |  |  |
|  |  | Familiarity with surgery speeds process | Use when expressing positive experiences of patients involving knowledge/familiarity with how to navigate care due to prior successful experiences with surgery |
|  |  | Learn from others' experiences with hernia brings comfort | Use when expressing positive experiences of patients involving when patient uses another person's experience with hernia to guide own care path. DO NOT use when getting advice from people without hernias (see: “others influencing care”) |
|  |  | Religion/faith as comfort | Use when expressing positive experiences of patients involving faith helping to address patient concerns or fears of the unknown outcomes of care for hernia |
|  |  | NHIF offers financial security | Use when expressing positive experiences of patients involving successful protection from financial costs involved in hernia care through NHIF coverage |
|  |  | Trust in NHIF for financial protection | Example of patient believing NHIF will provide protection for costs for care. DO NOT use if NHIF has ALREADY provided protection (see: “NHIF provide protection”) |
| Family/community support | Reaching, receiving |  |  |
|  |  | Family support financially | Use when expressing positive experiences of patients involving family fundraising or helping to cover medical OR non-medical expenses for patients regarding their hernia care |
|  |  | Family/community provide social support | Use when expressing positive experiences of patients involving examples of family assistance/support for patients with encouragement or company to avoid feelings of isolation or helplessness. DO NOT USE when referring to family influencing care seeking decisions (see: “family influence care seeking”) |
|  |  | Family support in tasks/function | Use when expressing positive experiences of patients involving family assistance when patient is unable to work or perform daily tasks due to hernia disease or during post-op recovery |
| Provider delays to care | Receiving |  |  |
|  |  | Scheduling/hospital | Use when patients experience delays stemming from HOSPITAL causes which are out of their control (ex: delayed appointments, theatre dates, etc) |
|  |  | Incorrect diagnosis | Use when patients are misdiagnosed with another cause for symptoms which leads them down incorrect treatment pathway until symptoms recur. Often out of patient control and associated with provider |
| Experience undergoing surgery includes… | Receiving |  |  |
|  |  | No pre-op preparation | Use when patients report LACK of specific preparations before undergoing hernia repair. Do NOT use with general emotions towards receiving general care in hospital |
|  |  | Pre-op preparations | Examples of specific preparations patients complete PRIOR to undergoing hernia repair. May include activities at home, notifying family, etc |
| Impact of operation | Receiving |  |  |
|  |  | Positive impact of surgery | Use when patients report positive emotions/lack of regret specifically towards outcome of surgery (ex: regained function, etc), even beyond the period of hospitalization. Do NOT use when referring to general satisfaction about quality of care received at MTRH (see: “satisfied with care”) |
|  |  | Post-op complications | Use when patients report experiencing post-op complications during recovery following hernia repair |
| Satisfied with care at MTRH | Receiving |  |  |
|  |  | Recommend MTRH for future | Use when patients express satisfaction or positive emotions towards the care they received at MTRH, leading to patients expressing that they or others should seek care at MTRH for any future problems which may arise |
|  |  | Satisfied with attention at MTRH | Use when patients express satisfaction or positive emotions towards the care they received at MTRH, leading to patients feeling that attention received from providers at MTRH (ex: rounding teams, nursing, etc) was satisfactory |
|  |  | Satisfied with clinical care at MTRH | Use when patients express satisfaction or positive emotions towards the care they received at MTRH, leading to patients feeling that general care received at MTRH (ex: experience undergoing surgery, symptom management, etc) was satisfactory. Do NOT use when expressing long-term positive feelings towards undergoing surgery (see: “impact of surgery”) |
| Dissatisfied with care at MTRH | Receiving |  |  |
|  |  | Inadequate attention | Use when patients express dissatisfaction/negative emotions regarding inadequate attention from providers at MTRH (ex: too busy, inadequate teaching, etc) while receiving care. More related to feeling cared/provided for |
|  |  | Inadequate teaching at MTRH | Use when patients express lack of teaching from providers regarding information they would like to know (ex: hernia disease, surgery procedure, post-op instructions). This may include patients NOT being able to reproduce information, even if they may have been given it before |
|  |  | Inadequate care | Use when patients express dissatisfaction/negative emotions regarding inadequate care from providers (ex: inappropriate symptom control, not addressing patient concerns, etc). More related to clinical issues |
|  |  | Unclear communication | Use when patients express dissatisfaction/negative emotions regarding inadequate communication with providers (ex: timing of surgery, reason for clinic visits, etc). More related to administrative issues |
|  |  | Poor ward environment at MTRH | Use when patients express dissatisfaction/negative emotions regarding inadequate or uncomfortable environment in ward during admission or recovery |
